# Radiotherapy of nasopharyngeal cancer using Rapidarc: dosimetric study of military teaching hospital Mohamed V, Morocco

**DOI:** 10.1186/s13104-017-2430-2

**Published:** 2017-02-28

**Authors:** Issam Lalya, El Amin Marnouche, Maghous Abdelhak, Noha Zaghba, Khalid Andaloussi, Mohamed Elmarjany, Laila Baddouh, Keltoum Dahmani, Khalid Hadadi, Hassan Sifat, Hamid Mansouri

**Affiliations:** 1 Department of Radiotherapy, Mohamed V Military Hospital, Rabat, Morocco; 2 Cadi Ayyad University, Marrakech, Morocco

**Keywords:** Nasopharyngeal cancer, Volumetric-modulated arc therapy, Intensity-modulated radiotherapy

## Abstract

**Background:**

The aim of the present study is to assess efficacy and efficiency of Volumetric-modulated arc therapy (VMAT) technique in treatment of nasopharyngeal cancer in our institution and to report toxicity related to this technique.

**Methods:**

Between June 2013 and January 2015, thirty-two patients with non metastatic nasopharyngeal cancer were curatively treated using VMAT Rapidarc. Dose prescription was performed using two different schedules, it consisted of either simultaneous integrated boost or simultaneous modulated accelerated radiation therapy delivering 70 Gy in 35 fractions and 69.96 in 33 fractions respectively. The choice was leaved at the discretion of the treating physician. The optimization process was performed by Eclipse software version 10.0 (Varian Medical Systems), using PRO algorithm (Progressive resolutive optimisation) version 3. Data was collected from dose-volume histograms for both planning target volumes (PTV) and organs at risk (OAR). We calculated the homogeneity index and the conformity index as well as the number of monitor units MU and the treatment delivery time. We also reported acute and late toxicity related to radiation therapy.

**Results:**

For the PTV high risk (HR), intermediate risk (PTV IR) and low risk (LR) the D95% was 97.21 ± 1.5, 97.5 ± 3.3 and 97.10 ± 6.86 respectively. Whereas, The D5% was 104.6 ± 2.16, 103.8 ± 2.1 and 100.89 ± 7.26. The CI for PTV HR was 0.98 ± 0.02 and the HI was 0.08 ± 0.02. The mean treatment delivery time was 2.3 ± 0.2, and the mean MU number was 527.6 ± 131.4. Grade 4 toxicity was not reported in any case. Grade 3 xerostomia was observed in only 3(9.4%) patients and no patients developed grade 3 hearing loss.

**Conclusion:**

Our results confirmed the abilities of VMAT to provide excellent coverage of target volumes while sparing OAR especially the nervous structures and salivary glands.

## Background

There have been significant advances in the delivery of radiotherapy. These include increased improvement of imaging techniques, which has resulted in improved accuracy of target volume definition and delineation, as well as sophistication in treatment planning systems and linear accelerator delivery capabilities leading to improved dose distributions and conformity. Volumetric-modulated arc therapy (VMAT) is also a major advance in the radiotherapy field. It is in an arc-based approach of intensity-modulated radiotherapy (IMRT) that allows irradiation with simultaneously changing multileaf collimator (MLC) position, gantry position, and dose rate. These abilities are extremely interesting when treating nasopharyngeal cancer; as its location and the proximity of the surrounding organs at risk (OAR) make radiation delivery very tricky.

The aim of this study is to assess efficacy and efficiency of VMAT technique in treatment of nasopharyngeal cancer in our institution and also to report radiation related toxicity when this technique is used.

## Methods

### Study population

Our study is a dosimetric retrospective one including 32 patients who were curatively treated, between June 2013 and January 2015, for intermediate (stage II), and advanced (stage III, IVA, IVB) nasopharyngeal cancer (NPC). Of note, patients where staged according to the tumor node metastasis (TNM) system of the International Union against Cancer (UICC) and American Joint Committee on Cancer (AJCC) 7th edition. All patients received concurrent chemoradiotherapy and some of them received previously induction chemotherapy. Radiotherapy (RT) was delivered using VMAT technique (rapidarc). Informed consent (verbal) was obtained from all participants. This study was submitted to and approved by research and ethics committee of military teaching hospital Mohamed V.

### Treatment planning

Computed tomography (CT) datasets were taken from a CT simulator (CT Simulator, General Electric, Cleveland, OH) using a 2.5 mm slice thickness. Intravenous contrast was routinely used unless contraindicated. All patients were immobilized using 5 points mask in supine position.

Target volumes delineation, using CT and magnetic resonance imaging (MRI), was as follows; the gross tumour volume (GTV), including the macroscopic primary cancer and nodes greater than 1 cm in short axis or nodes with necrotic centres, was delineated. Three clinical target volumes CTV according to dose levels were defined for each patient; the CTV HR (high risk) is defined using an isotropic margin of 5 mm around the GTV and is extended to include the entire nasopharynx. The CTV IR (intermediate risk) is defined as the volume including the CTV HR with a 3 mm margin around plus areas at risk for microscopic involvement; including skull base (ensuring coverage of foramen ovale), anterior half of clivus (including entire clivus if involved), lower half of sphenoid sinus (in T3, T4 disease include entire sphenoid sinus), pterygoid process, pterygopalatine fossae, parapharyngeal and retro-styloid spaces, retropharyngeal nodes, nodal levels containing lymphadenopathy and their adjacent ones. The CTV LR (low risk) is delineated by adding an isotropic margin of 3 mm around the CTV IR and then extended to include the posterior third of the nasal cavity/maxillary sinuses, and nodal levels (level II to V). Safety margin between the CTV and planning target volume (PTV) was fixed at 3–5 mm for all CTVs to account for patient set-up and motion uncertainties, but in areas in which the GTV or the CTV was adjacent to critical normal structures (i.e. the brainstem) the margin was reduced to 1 mm.

Organs at risk were also delineated, it included organs as follows spinal cord, brain stem, chiasma, optic nerves, lens, eyes, inner ear, parotid and submandibular glands and temporo-mandibular joints.

Dose prescription was performed according to two schedules; Simultaneous integrated boost (SIB) or simultaneous modulated accelerated radiation therapy (SMART). The choice of either one was leaved at the discretion of the treating physician. In the SIB treatment modality, the PTV HR, IR, LR received respectively 70 Gy at 2 Gy/fraction, 63 Gy at 1.8 Gy/fraction, 56 Gy at 1.6 Gy/fraction in 35 fractions. Whereas in the SMART modality,the PTV HR, IR, LR received 69.96 Gy at 2.12 Gy/fraction, 59.4 Gy at 1.80 Gy/fraction, and 54 Gy at 1.64 Gy/fraction in 33 fractions respectively. Patients were irradiated once a day, five times a week.

The optimization process, as part of inverse planning, was performed by Eclipse software version 10.0 (Varian Medical Systems), using PRO algorithm (Progressive resolutive optimisation) version 3.

### Evaluation parameters

Data was collected from dose-volume histograms (DVH) for both PTV and OAR. For the PTV, evaluation parameters included PTV volume, D2% (relative dose absorbed by 2% of the PTV), D5% (relative dose absorbed by 5% of the PTV), D95% (relative dose absorbed by 95% of the PTV), D98% (relative dose absorbed by 98% of the PTV), V95% (volume surrounded by the isodose 95% of the prescribed dose).According to the radiation therapy oncology group (RTOG) guidelines [1], we calculated the homogeneity index (HI) as follows: HI2 = D2%/D98%,or HI5 = D5%/D95%. The HI could also be obtained otherwise; HI* = (D2–D98%)/D50%. Conformity index (CI) was also calculated using the following formula: CI = V95%/PTV HR. Ideally HI2, HI5 and CI should be equal to 1, whereas HI* should be equal to 0. For OAR, we reported Dmax for spinal cord, brain stem, chiasma, optic nerves, temporo-mandibular joints. For parotid and submandibular glands, we reported Dmean, V30 and V40 (the percent of gland receiving ≥30 Gy, 40 Gy).

The treatment time and Monitor Units (MU) were extracted from the patient’s electronic medical record in ARIA^®^ to assess treatment delivery efficiency.

### Toxicity assessment

All patients had weekly clinical evaluation during the treatment. Patient’s follow-up was continued every 3 months for the first 2 years, then every 6 months for years the next 3 years and yearly thereafter.

Toxicity was graded according to the Common Toxicity Criteria for Adverse Events (CTCAE v4.03), evaluating the oral mucositis, xerostomia, dermatitis, hearing loss and brain necrosis. Toxicity was defined as acute when occurring during radiotherapy or within the first 6 months after the end of radiotherapy. Late toxicity was defined as toxicity occurring beyond 6 months after the end of radiotherapy.

### Statistical analysis

A Statistical Package for Social Sciences package (SPSS 20.0) was used for data processing. The normal distribution of variables was preliminarily checked with the kolmogorov–smirnov test, and then expressed in mean and standard deviation. To determine influencing factors, we used Pearson test “r” to assess correlation and linear regression analysis. Each test would have to be significant with a p value < 0.05.

## Results

### Patient characteristics

Table 1 summarizes patient characteristics. Of note, Three quarters of patients were staged as III and IV.

**Table 1:** Patient characteristics

Characteristics	Results
Age^a^	44.75 ± 14.63
Gender^b^	
Men	11 (34.4)
Women	21 (65.6)
TNM stage^b^	
I	0
II	7 (21.9)
III	15 (46.9)
IVA	8 (25)
IVB	2 (6.3)

^a^Quantitative variable expressed in mean ± SD (standard deviation)

^b^Qualitative variable expressed in number (n) and percentage (%): n (%)

### Target coverage, homogeneity and conformity index

All PTV (high risk HR, intermediate risk IR and low risk LR) received doses ranging from 95 to 107% of the prescribed dose. The D98%, also called near-minimum absorbed dose, was 94.74 ± 3.4, 95 ± 3.9 and 92.67 ± 14.56 for PTV HR, IR and LR respectively. While, the D95% was, for the same volumes, 97.21 ± 1.5, 97.5 ± 3.3 and 97.10 ± 6.86 respectively. The D2%, also called near-maximum absorbed dose, was respectively 105.17 ± 2.32, 104.47 ± 2.2 and 101.84 ± 7.08.

The homogeneity index was very close to 1(1.08 ± 0.02 for PTV HR, 1.22 ± 0.05 for PTV IR and 1.5 ± 0.81 for PTV LR). So was the conformity index (0.98 ± 0.02). Parameters coverage of PTV was summarized in Table 2.

**Table 2:** Summary of dose coverage of the PTVs

Target volume	Parameter	Objective	Mean ± SD
PTV HR	PTV HR (cm^3^)		241.11 ± 144.6
	D2%		105.17 ± 2.32
	D5%		104.6 ± 2.16
	D95%		97.21 ± 1.5
	D98%		94.74 ± 3.4
	V95% (cm^3^)		236.32 ± 142.38
	HI2	1	1.08 ± 0.02
	HI5	1	1.08 ± 0.02
	HI	0	0.08 ± 0.02
	CI	1	0.98 ± 0.02
PTV IR	VPTV IR (cm^3^)		488.51 ± 252.12
	D2%		104.47 ± 2.2
	D5%		103.8 ± 2.1
	D95%		97.5 ± 3.3
	D98%		95 ± 3.9
	V95% (cm^3^)		316.35 ± 218.72
	HI2	1	1.22 ± 0.05
	HI5	1	1.18 ± 0.04
PTV LR	VPTV LR (cm^3^)		667.15 ± 335
	D2%		101.84 ± 7.08
	D5%		100.89 ± 7.26
	D95%		97.10 ± 6.86
	D98%		92.67 ± 14.56
	V95% (cm^3^)		269.17 ± 201.2
	HI2	1	1.5 ± 0.81
	HI5	1	1.3 ± 0.16

### Organs at risk

Dose limits were respected in all nervous structures (spinal cord, brainstem…). However, the parotid absorbed dose was above the dose limits for Dmean, V30 and V40, for both parotid glands. The PTV IR was significantly correlated to contralateral parotid Dmean and V30 with p = 0.002 and p = 0.027 respectively (Table 3). In simple linear regression, the extent of PTV IR influenced significantly both Dmean and V30 of contralateral parotid with p = 0.016 and p = 0.027 respectively. Results concerning OAR doses are reported in Table 4.

**Table 3:** Correlation between PTV volumes and parotid parameters

	Contralateral parotid			Ipsilateral parotid		
	Dmean	V30	V40	Dmean	V30	V40
PTV HR	0.059	0.075	0.283	0.120	0.265	0.212
PTV IR	0.002*	0.027*	0.941	0.052	0.491	0.948
PTV LR	0.182	0.135	0.838	0.170	0.280	0.233

* p < 0.05

**Table 4:** Summary of OAR doses

OAR	Parameter	Dose constraints^a^	Mean ± SD
Spinal cord	Dmax (Gy)	≤45 Gy	43.84 ± 3.68
Brainstem	Dmax	≤54 Gy	54.65 ± 2.8
Chiasma	Dmax	<54 Gy	41.67 ± 22.88
Optic nerve			
Ipsilateral	Dmax	<54 Gy	42 ± 19.24
Contralateral			41.26 ± 18.68
Parotid gland			
Ipsilateral	Dmean (Gy)	≤26 Gy	42.66 ± 8.83
	V30 (%)	<50%	74.47 ± 24.04
	V40 (%)	≤33%	49.71 ± 25.54
Contralateral	Dmean		39.77 ± 9.73
	V30 (cm^3^)		66.01 ± 27.01
	V40(%)		41.92 ± 23.88
Submandibular gland			
Ipsilateral	Dmean		7.19 ± 2.45
Contralateral	Dmean		7.25 ± 2.25
Inner ear			
Ipsilateral	Dmax	<50 Gy	59.12 ± 8.31
Contralaetral			57.32 ± 8.19
Temporo-mandibular joint			
Ipsilateral	Dmax	<60 Gy	62.01 ± 7.77
Contralateral			58.26 ± 7.08

^a^RTOG protocol 0225

### Acute and late toxicity related radiotherapy

With a follow up duration of 19.78 ± 4.3 month, all patients were healthy; no grade 4 toxicity was reported. Grade 3 xerostomia was observed in only 3 (9.4%) patients and no patients developed grade 3 hearing loss (Table 5).

**Table 5:** Acute and late toxicity related to radiation treatment

	Acute toxicity (grading)^a^					Late toxicity (grading)^a^				
	no	1	2	3	4	no	1	2	3	4
Radiation mucitis	4 (14.8)	11 (40.7)	11 (40.7)	1 (3.7)	0					
Radiation dermatitis	1 (3.7)	4 (14.8)	20 (74.1)	2 (7.4)	0					
Xerostomia						5 (15.6)	15 (46.9)	9 (28.1)	3 (9.4)	0
Hearing loss						18 (56.3)	10 (31.3)	4 (12.5)	0	0
Brain radiation necrosis						32 (100)	0	0	0	0

^a^Qualitative variable expressed in n (%)

### Treatment efficiency

**Fig. 1:**
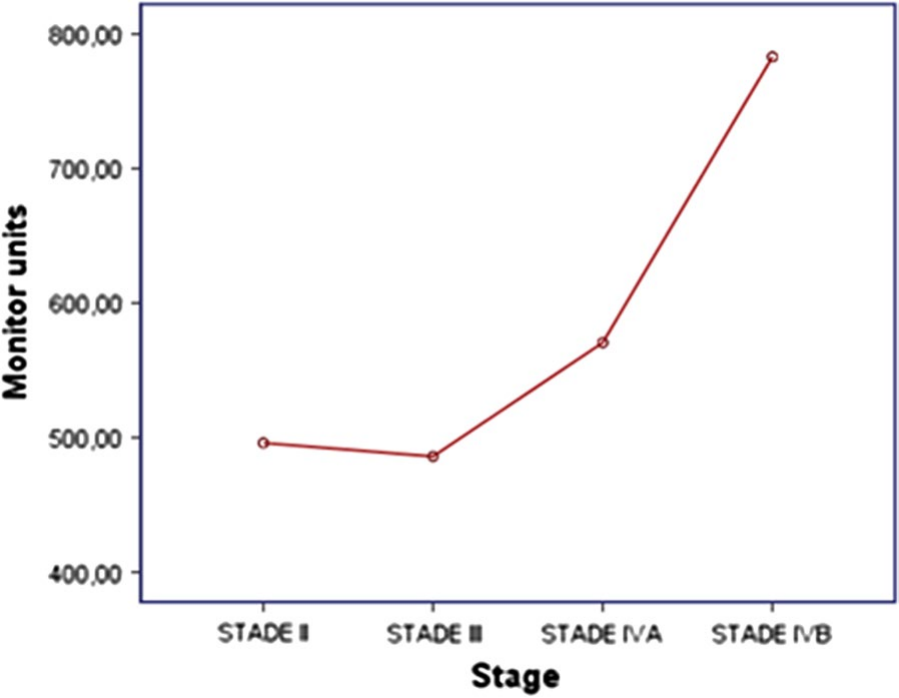
Average of MU reported by TNM stage

**Fig. 2:**
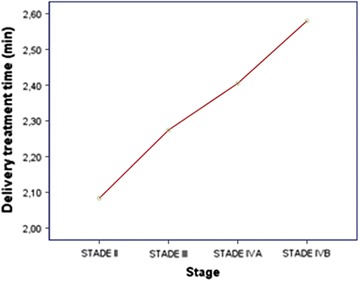
Average of delivery treatment time reported by stage

Double arc VMAT plan was achieved for all patients. Both number of MU and delivery treatment time increased as the disease stage worsened (Figs. 1, 2). In simple linear regression, stage has a statistically significant relationship with MU (p = 0.007) and treatment delivery time (p = 0.002). Results regarding delivery treatment time and MU are summarized in Table 6.

**Table 6:** Treatment efficiency

	Mean ± SD
MU	527.6 ± 131.4
Delivery treatment time (min)	2.3 ± 0.2

## Discussion

Volumetric-modulated arc therapy is an arc-based approach of IMRT that allows irradiation while changing the dose rate, MLC and gantry position [2, 3]. This technique intends to resolve some of the major limitations of fixed field IMRT (large number of MU and treatment delivery time increased). NPC is a good example to evaluate VMAT abilities because of the location of the nasopharyngeal region that is at a more superior level when compared to other head and neck cases. Also, target volumes are surrounded by a relatively larger volume of organs at risk such as neurologic structures (brain stem, spinal cord, optical nerves), and parotid glands.

To evaluate target volumes coverage, there are many parameters such as D2%, D98%, and homogeneity, conformity index, which are recommended by the ICRU83 report. However, there is no standardized formula to calculate CI and HI. This issue makes difficult any comparison between different series especially for CI. Moreover, most studies evaluate VMAT on head and neck cancers and only few evaluate VMAT in only NPC. Lee et al. [4] reported, in their series, a HI value of 1.07 ± 0.01 which is comparable to our study (1.08 ± 0.02). Using another formula to evaluate HI (An HI of zero indicates that the absorbed-dose distribution is almost homogeneous) Guckenberger et al. [5] reported 0.07 versus 0.08 in our study.

As to OAR, IMRT has proven to be effective in reducing the dose to some critical adjacent organs (spinal cord, brainstem, temporal lobe, parotid glands, optic chiasm, and mandible,) when compared to conventional RT [6, 7]. Using the VMAT, the proportion of OAR spared is at best slightly better when compared to fixed field IMRT [8, 9]. The major concern when irradiating NPC is the brainstem and the parotid glands. In this study, the average maximum dose to the brain stem was 54.65 ± 2.8 Gy which exceeds the dose constraints of 54 Gy but only by a small amount. In fact, the Quantitative Analyses of Normal Tissue Effects in the Clinic (QUANTEC) recommendations states that the entire brainstem may be treated to 54 Gy using conventional fractionation with limited risk of severe or permanent neurologic effects. Partial volumes of the brainstem (1–10 mL) may be irradiated to a maximum dose of 59 Gy for dose fractions ≤2. Parotid glands’ sparing is the another benefit of IMRT and VMAT to avoid xerostomia. In fact, the mean parotid gland dose appears to be associated with gland salivary production [10, 11]. Minimal reduction in flow is observed at mean doses <10–15 Gy, decreases gradually over the range of 20–40 Gy, and is markedly reduced above 40 Gy [10, 12]. Consequently, the risk of xerostomia is reduced when at least one parotid gland or submandibular gland is spared [13]. Portaluri et al. [14] reported that a dose <30 Gy to the contralateral parotid results in mild subjective xerostomia or no xerostomia at all. Unfortunately, in our study, Dmean in contralateral parotid was 39.77 ± 9.73, and only 2(6.3%) and 4(12.5%) patients had Dmean in contralateral parotid <26 Gy and <30 Gy respectively. These results might be explained by the fact that 75% of patients had locally advanced stages (stage III and IV). Our results are concordant with those presented by Zheng et al. [15], as they recorded Dmean in left and right parotid of 32.9 ± 7.5 and 33.4 ± 9.8 Gy respectively. In their study of 20 patients, 13 patients were stage III and 7 patients were stage IV. Nevertheless, after a follow up duration of 19.78 ± 4.3 month, we did not record any grade4 toxicity and grade 3 xerostomia was observed in only 3(9.4%) patients. These early results suggest that the parotid gland can tolerate more absorbed dose and that dose limits may be revised.

The main disadvantage of the VMAT is the increased time to generate the VMAT plans, which can be explained by the large number of beams (control points) involved in planning for VMAT, or by the need of a learning curve to adapt to this new technique. However, the planning time may be reduced with the continued optimization of VMAT planning techniques and increased computer hardware and software processing speed [16]. In general, more than one arc is required to generate an acceptable dose distribution because of the complexity of the target volumes in head and neck radiotherapy. Guckenberger et al. [5] found, in a retrospective planning study comparing step-and-shoot IMRT with VMAT for 20 patients that two and three VMAT arcs allowed similar outcomes compared to IMRT in postoperative and primary radiotherapy for pharyngeal cancer. Other planning studies confirmed the advantage of two arcs compared to a single arc in terms of PTV coverage and OAR sparing [17, 18]. In our study, all treatment plans were delivered with two arcs.

Available data on IMRT suggests that IMRT plans require a higher number of monitor units (MU) compared to conventional radiotherapy (CRT) plans and probably increase the proportion of low dose radiation to the rest of the body. In fact, the number of MU in an IMRT plan may be three times higher than a CRT plan leading to an increase in the incidence of radiation induced secondary malignancies from 1 to 1.75% in patients surviving 10 years or more after treatment [19]. Moreover, as IMRT plans often require multiple fixed angle radiation beams, treatment delivery time is often increased when using this technique. This could compromise the reproducibility of treatment position patient, and could also have radiobiological consequences allowing the possibility of increased tumor cell repair and repopulation [20, 21]. VMAT has attracted particular attention giving its ability to provide highly conformal dose distribution and good delivery efficiency while reducing treatment delivery time. Lee et al. [4] have found that the mean delivery time of 8.2 ± 0.4 min in 7F IMRT was significantly reduced by 51 to 41% when a single arc (4.0 ± 0.6 min) or double arc (4.8 ± 0.4 min) was respectively used, leading to a reduced number of MU. In fact, the mean values of MU/fraction were 773 ± 48 in 7F IMRT, 445 ± 33 in single arc and 493 ± 36 in double arc; this can be translated to a 42 and 36% reduction in monitor units/fraction when compared to 7F IMRT, allowing a reduction of low dose radiation to the rest of the body. Vanetti et al. [8] have confirmed this finding, in their study; VMAT plans achieved a reduction of the integral doses to the body by an average of 7% when compared to the fixed field IMRT plans. This is extremely interesting as it allows reducing the risk of secondary radiation-induced malignancies, which is of major concern in pediatric patients or patients with long life expectancies [19]. In our study, treatment delivery time was 2.3 ± 0.2 min and the mean number of MU was 527.6 ± 131.4. More advanced stage needed higher number of MU and more time to be delivered.

## Conclusion

Our results confirms the abilities of VMAT to provide excellent coverage of target volumes and sparing OAR as much as possible especially for nervous structures and salivary glands without sacrificing the delivery efficiency.

## Abbreviations

VMAT: Volumetric-modulated arc therapy
IMRT: intensity-modulated radiotherapy
MLC: multileaf collimator
OAR: organs at risk
NPC: nasopharyngeal cancer
TNM: tumor node metastasis
UICC: International Union against Cancer
AJCC: American Joint Committee on Cancer
RT: Radiotherapy
CT: computed tomography
MRI: magnetic resonance imaging
GTV: gross tumour volume
CTV: clinical target volumes
PTV: planning target volume
HR: high risk
IR: intermediate risk
LR: low risk
SIB: simultaneous integrated boost
SMART: simultaneous modulated accelerated radiation therapy
PRO: progressive resolutive optimization
DVH: dose-volume histograms
RTOG: radiation therapy oncology group
HI: homogeneity index
CI: conformity index
MU: Monitor Units
CTCAE: Common Toxicity Criteria for adverse event
SD: standard deviation
